# Can machine learning help accelerate article screening for systematic reviews? Yes, when article separability in embedding space is high

**DOI:** 10.1017/rsm.2024.16

**Published:** 2025-03-10

**Authors:** Farhan Ali, Amanda Swee-Ching Tan, Serena Jun-Wei Wang

**Affiliations:** 1 National Institute of Education, Nanyang Technological University, Singapore, Singapore; 2 College of Computing and Data Science, Nanyang Technological University, Singapore, Singapore

**Keywords:** active learning, embedding large, language models, machine learning, systematic reviews

## Abstract

Systematic reviews play important roles but manual efforts can be time-consuming given a growing literature. There is a need to use and evaluate automated strategies to accelerate systematic reviews. Here, we comprehensively tested machine learning (ML) models from classical and deep learning model families. We also assessed the performance of prompt engineering via few-shot learning of GPT-3.5 and GPT-4 large language models (LLMs). We further attempted to understand when ML models can help automate screening. These ML models were applied to actual datasets of systematic reviews in education. Results showed that the performance of classical and deep ML models varied widely across datasets, ranging from 1.2 to 75.6% of work saved at 95% recall. LLM prompt engineering produced similarly wide performance variation. We searched for various indicators of whether and how ML screening can help. We discovered that the separability of clusters of relevant versus irrelevant articles in high-dimensional embedding space can strongly predict whether ML screening can help (overall *R* = 0.81). This simple and generalizable heuristic applied well across datasets and different ML model families. In conclusion, ML screening performance varies tremendously, but researchers and software developers can consider using our cluster separability heuristic in various ways in an ML-assisted screening pipeline.

## Highlights

### What is already known

- Systematic reviews can be very time-consuming, necessitating the need for semi-automated procedures involving machine learning (ML) strategies.
- The specific strategy of active ML shows promise at the screening stage but whether and how it can help remains unclear.
- Large language models (LLMs) have emerged, but there is a lack of systematic investigation on their potential for use in systematic reviews.

### What is new

- We found a huge variation in ML screening performance across datasets using classical machine learning, deep learning, and LLM model families.
- We discovered a simple exponential function to explain this performance variation based on separability of clusters of relevant versus irrelevant articles in high-dimensional embedding space.

### Potential impact for research synthesis methods readers

- Cluster separability provides a unifying explanation of the varied performance in ML-assisted systematic reviews.
- Our cluster separability heuristic can be used in ML-assisted pipeline at the stages of model selection, search and inclusion criteria, and stopping decision.

## 1 Introduction

Systematic reviews play important roles in many fields. They consolidate and summarize research findings in a transparent and unbiased manner, provide the basis for evidence-based practices and interventions such as via subsequent meta-analysis, and can highlight current gaps and conflicts as well as future research directions.^1^ ^–^ ^3^ However, systematic reviews can be very time-consuming, estimated to take thousands of hours spanning over a year.^4^ ^,^ ^5^ This challenge is compounded by the rapid recent growth in the scientific literature^6^ including in education.^7^ Moreover, with changing demands and situations such as COVID-19, systematic reviews need to serve pressing needs in a timely manner.^8^ Overall, it is important to develop and evaluate automated strategies to accelerate systematic reviews.

The systematic review process involves many aspects, often times formalized and reported via the Preferred Reporting Items for Systematic Reviews and Meta-Analyses (PRISMA) framework.^9^ The most pertinent aspect of PRISMA for the present study involves the search, screening, and eligibility assessment of articles. In the first stage, databases are searched using keywords to identify articles, often numbering in the thousands. From this full set of articles, a screening of the abstract identifies a subset of potentially relevant articles that would then undergo a more thorough full-text assessment for final inclusion in the review. This multistage filtering can involve complex inclusion and exclusion criteria to differentiate relevant from irrelevant articles. It also involves various challenges such as inconsistency between screeners, fatigue and errors. Can machine learning (ML) help assist this screening workload? In this study, we addressed this general question.

The rise of ML has led to its incorporation to aid systematic reviews.^10^ Active ML may be particularly useful. It is a form of ML in which data points are “actively” selected as part of the learning process rather than using all or random selections of data points. In a common human-in-the-loop implementation, a human provides initial labels of some relevant and irrelevant articles that the model then learns from. After multiple iterations, the model learns to more efficiently identify the relevant articles which a human can further verify, reducing the need to go through the rest of the unseen record of articles that are potentially irrelevant. The use of active ML has been incorporated into various tools^11^ ^,^ ^12^ and has shown good results across a wide range of fields. In a recent large-scale study involving over 160,000 articles across 26 datasets, active ML offered, on average, 62–71% of work saved at 95% recall.^13^ But this average masked a great variation of performance across datasets from just above 0% to ~90% work saved. It is currently unknown why such variations exist, an important problem in the field of automated screening.^13^

One hypothesis to explain the variation is that some datasets are just “harder” to classify, regardless of the models being used, due to characteristics inherent to the dataset itself. For example, some datasets have a very large full set (thousands of records) but a very small relevant article set (just a few) which would truly stump any supervised ML model to learn to classify due to the extreme imbalance. Another characteristic might be the semantic content of the relevant and irrelevant articles. Individual terms, phrases, and constructs that are found in the irrelevant articles may also be found in the relevant articles which would make it harder for supervised ML models to separate the relevant from irrelevant articles. One possibility is the use of high-dimensional text representations that can go beyond single words and incorporate more complexities of contexts and correlations, allowing better separation of the relevant from irrelevant articles. This idea is reminiscent of a kernel trick in ML models. In this sense, how separable the distribution of relevant articles is from the irrelevant articles in high-dimensional semantic text representations may give us a clue as to how well any downstream supervised learning model may perform for that dataset. To our knowledge, there is no systematic investigation of whether and how dataset characteristics including the notion of article separability may explain variation in ML screening performance.

In implementing active ML, there is a wide selection of models to choose from. Classical ML models such as logistic regression and support vector machine have been utilized with promising, though highly varied, performances as reviewed above. However, these classifiers are considered shallow in model architecture and may be unable to identify more complex relationships in input space. For systematic reviews, the input text space may exhibit certain complexities that shallow classifiers may not be able to leverage for improved performance. For example, these classifiers typically use text representations that assume individual words as being independent and are limited in incorporating sequence information, context of words, and more complex text correlations. Deep neural networks can overcome these limitations given their deep, multilayer architecture that can automatically learn local, global, and hierarchical text correlations. Teijema et al.^14^ recently pioneered the use of convolutional neural networks (CNNs) in automated screening, finding evidence of improved performance compared to classical models. This improvement was seen especially when shallow models were used at the beginning to generate initial labels before switching to CNNs. This beneficial switching effect is hypothesized to be due to the benefit of sufficient labels from non-CNN models to initialize and train the large parameter space for CNNs, and the power of CNNs to identify hard records, especially the last ones, in complex semantic clusters. However, some of the performance benefits post-switching were small or nonexistent when compared to different classical models While promising, there is a need for a systematic test of deep neural networks on additional datasets.

Large language models (LLMs) have recently emerged as powerful versatile tools with potential for use in systematic reviews. In particular, few-shot learning via prompt engineering has exhibited very impressive performances in a wide variety of domains.^15^ ^,^ ^16^ This strategy does not involve the typical ML training such as via changes to neural network weights, which can be rather costly and data-intensive. Instead, the strategy uses natural language prompts as inputs to steer LLM outputs. It leverages the complex language capabilities of LLMs and is believed to be a latent property of very large neural networks.^15^ ^,^ ^17^ Prompts can be configured to include shots (examples) of inputs (e.g., article titles and abstracts) and outputs (classification of relevant or irrelevant) in order to provide more information for LLMs to classify the queried article. While LLMs may show promise, there might be challenges. The vast majority of LLMs are general purpose and not fine-tuned to academic research; thus, their performance on academic texts may be suboptimal. LLMs also currently have limited context length, meaning it might not be feasible to do few-shot learning where article texts are very long. Prompts including shots can reach thousands of tokens (pieces of words), nearing the limits of numerous LLMs. While newer LLMs are being released with larger context lengths, accurately interpreting such long and complex prompts remains a challenge.^18^ The use of LLMs including via few-shot learning have recently been tested on systematic review datasets in multiple fields.^19^ ^–^ ^21^ However, there is a lack of systematic investigation using various datasets while variances in performance have been unexplained.

A review of the literature suggests the educational field as a good context to investigate ML screening strategies. Educational research involves terms, phrases, and constructs that may not be consistent and clear enough for ML models to learn well from unlike, for example, in medicine where there are highly standardized taxonomies of terms, conditions, symptoms, and so forth^22^ Moreover, inclusion and exclusion criteria can be complex and lacking consistency in terms, phrases and constructs, further questioning whether ML models can learn to screen educational articles. Two recent studies are among the first to investigate the potential for ML screening in education. In one study, four ML models were applied to one dataset, finding about 70% work saved.^23^ In another, Campos et al.^22^ employed active ML in the software ASReview on a relatively large dataset of 27 educational and educational psychology reviews. They found, on average, 58% work saved, lower than the average performance found across other fields.^13^ ^,^ ^24^ However, both studies in educational research used a limited set of classical ML models without any deep learning or emerging LLMs. Nor was variation in performance across datasets explained. Nonetheless, these important early results suggest the promise of ML screening for educational reviews. Moreover, the use of screening tools including automated screening is generally quite low in educational research,^25^ potentially due to lack of knowledge or skepticism of ML performances and biases. This stands in contrast to wide use in other fields such as the medical sciences.^26^ Because of the strong reliance on evidence synthesis in education,^1^ a systematic investigation of ML’s potential is warranted.

In the current study, we performed a comprehensive analysis of a large combination of ML models on actual datasets of systematic reviews in education. We trained classical and deep learning model families. Given the uniqueness of systematic reviews involving highly imbalanced data, we further utilized recent active learning strategies in which learned data are structured in order to rapidly identify the relevant articles. We additionally tested emerging LLMs via few-shot learning. We also attempted to explain whether and how ML screening strategies work. We addressed the following research questions:

1. How much work in article screening can be saved by using ML models—classical and deep learning—on educational datasets?
2. How does LLM prompt engineering perform in article screening on educational datasets?
3. What can explain variation in ML screening performance across datasets?

## 2 Methods

### 2.1 Datasets

We identified recent (past 5 years) systematic reviews based on literature search of research areas we were familiar with (special education needs, educational technology, social–emotional learning). Of authors from 77 reviews who were contacted via email, seven authors kindly responded with their datasets (9.1% response rate; Table 1). Each dataset generally had article titles and for some, the abstracts. The dataset also had labels of whether each article was relevant, thus included after screening stage, or irrelevant, hence excluded from further assessment. We cleaned the dataset by deduplicating and retrieving the missing titles and abstract information using Scopus application programming interface (API). Non-English language articles were excluded as well as articles for which we could not retrieve the titles and/or abstracts. Thus, Table 1 may not exactly match the reported numbers in the original paper. Because of the convenience nature of the selection and the relatively low author response rates, we do not claim the datasets to be representative of educational research. Nonetheless, the datasets do exhibit useful properties such as large ranges in size (661–6578 articles) and relevance rate (1.21–19.4%; Table 1). As shown in the results, the ML performances were also highly varied, all of which gave us opportunities to model and explain the large variations. We also added a benchmark dataset from a distinct but related field of psychiatry^32^ to directly compare to the educational datasets in our study.

**Table 1:** Details of systematic review datasets analyzed in the present study

	Number of articles	Number of relevant articles
References	in full set	in full set (relevance rate)
Cinquin et al.^27^	944	87 (9.21%)
Clark and Reuterskiöld^28^	906	11 (1.21%)
Oliveira et al.^29^	661	52 (7.87%)
Ruggeri et al.^30^	6578	160 (2.43%)
Toffalini et al.^31^	1031	200 (19.4%)
van de Schoot et al.^32^ (benchmark dataset)	6185	363 (5.87%)
van Hoorn et al.^33^	3848	349 (9.07%)
Ward et al.^34^	1842	49 (2.66%)

### 2.2 ML pipeline

Our ML models were either families of non-CNN or CNNs. For non-CNN family of models, we used a combination of hyperparameters of feature extraction technique, classifier, balance strategy, and query strategy as shown in Table 2. Feature extraction via embedding allows representations of the text in numerical forms for subsequent ML. They were either classical feature extractions (TF-IDF) or neural network-based (Doc2Vec, Sentence BERT). An additional embedding via long short-term memory (Embedding-LSTM) could only be used for LSTM classifiers. Classifiers were either shallow classifiers (random forest, support vector machine, logistic regression, 2-layer neural network) or deep learning classifiers (LSTM one-output base model, LSTM multiple-output with pooling model). For CNN-based models, we implemented switching CNN-based classifiers in which a deep convolutional neural network with 10–20 hidden layers were used for automated feature extraction and classification. We used Optuna Python package^35^ to tune for the optimal number of layers. However, initial iterations of the learning were dependent on feature extraction and embedding as above before switching to the CNN-based architecture, a switching strategy that was previously shown to improve performance in automated screening via active learning.^14^ The data are expectedly imbalanced (see Table 1 for relevance rate). However, in our supplementary analysis, different balancing strategies (e.g., undersampling) had little to no effect on the performance results (mean difference in performance = −0.6% when comparing all 3 balancing strategies within each dataset). Thus, to ensure feasibility of running so many different combinations, we implemented and reported simple balancing strategy for the main runs in which there was no balancing attempted for both non-CNN and CNN families of models. Finally, the whole pipeline relied upon active learning in which to-be-queried data are identified in a structured manner. Thus, there was an additional parameter of query strategy in terms of which articles to label and consider next in the learning. Similar to balancing strategies, supplementary analysis indicated very little effect on performance of different query strategies (mean difference in performance = −0.2% when comparing all five query strategies within each dataset). Consequently, we chose just one to fully implement for the main runs, that of mix sampling involving maximum (most likely to be relevant) and random sampling for both non-CNN and CNN families of models. A total of 26 models (14 non-CNNs, 12 CNNs) were learned for each dataset based on the combination of hyperparameters. 100 independent runs were implemented for each model and the results averaged. The API of ASReview v1.1^12^ was used along with other required packages such as scikit-learn and tensorflow.

**Table 2:** ML hyperparameters for non-CNN and CNN family of models

Non-CNN family		Balance	
Feature extraction	Classifier	strategy	Query strategy
– TF-IDF (tf)– Doc2Vec (do) – Sentence Bert (sb) – Embedding-LSTM (em)	– Random forest (rf) – Support vector machine (svm) – Logistic regression (lo) – 2-layer neural network (nn) – Long short-term memory-base (lstm-base) – Long short-term memory with pooling (lstm-pool)	– Simple (no balancing)	– Mixed (maximum + random)
CNN family			
Feature extraction	Initial classifier (before switching)	Balance strategy	Query strategy
– TF-IDF (tf) – Doc2Vec (do) – Sentence Bert (sb)	– Random forest (rf) – Support vector machine (svm) – Logistic regression (lo) – 2-layer neural network (nn)	– Simple (no balancing)	– Mixed: maximum + random

*Note*: For non-CNN models, not all combinations were possible. LSTM classifiers work exclusively with embedding-LSTM feature extraction and vice versa.

### 2.3 LLM prompt engineering pipeline

The use of LLM was also investigated. In this pipeline, we used prompt engineering for few-shot learning, which has shown promise as a versatile strategy in many different domains^15^ ^,^ ^16^ (i.e., there was no transfer learning or fine-tuning of LLMs). We carefully engineered prompts for each dataset based on the original authors’ description of inclusion and exclusion criteria. Supplementary Table 1 shows the final engineered prompt for each dataset. The prompt was then combined together with shots (examples) of relevant articles’ combined titles and abstracts, and shots of irrelevant articles’ titles and abstracts. We used 0, 2, 4, or 8 randomly selected shots (balanced across relevant and irrelevant articles). The combined prompt was then used by the LLM label other combined titles and abstracts. The LLM labels were compared against the actual labels. LLM prompt engineering pipeline was implemented using API of GPT-3.5 and GPT-4 LLMs with temperature of 1.0 (October to December 2023).

### 2.4 Performance metrics

For active ML using shallow and deep classifiers, we used the metric of work saved from sampling (WSS).^12^ It represents the percentage reduction in the number of articles needed to be screened by using active ML instead of screening at random. WSS is quantified at a given level of recall of relevant articles. We chose 95% recall as humans generally can retrieve ~82% (based on abstract alone) or ~95% (based on full text) of relevant articles,^36^ suggesting that 95% recall is as good as, if not better than, a human screener. To illustrate the WSS metric, assume a dataset of 1000 articles in which 100 articles have been prelabeled as relevant. If just 15% of the whole dataset (150 articles = 15% × 1000 articles) needs to be screened to correctly retrieve 95% of relevant articles (95 articles = 95% × 100 articles), then the WSS @ 95% value is 80% (95–15%). A random screening strategy is expected to produce 0% WSS @ 95% as 95% of the dataset will need to screened in order to identify 95% of the relevant articles, indicating no work saved (95–95%). Supplementary Figure 1 provides a graphical example of the WSS @ 95% metric quantification. For LLM prompt engineering, there is no reliance on active learning, thus WSS metric does not apply. Instead, we relied on traditional measures of classification success/failure. We used Mathews correlation coefficient (MCC) as it considers all cells in a confusion matrix, thus giving a fuller evaluation based on recall, precision, false positives, and false omissions, ideal for imbalanced datasets. MCC ranges from −1 (predicted labels perfectly opposite of actual label) to 1 (predicted label perfectly matching the actual label). MCC^2^ is similar to *R* ^2^ which is variance in the actual labels explained by the model’s labels.

To examine variation in ML screening performance, we modeled performance metrics as a function of various characteristics of the dataset as input variables such as original full set size, relevant set size, and relevance rate (% of articles that are relevant). In addition, we tested the idea that the separability of relevant and irrelevant articles in high-dimensional semantic space can determine ML screening performance. To this end, we embedded (100 dimensions) each article’s combined title and abstract using the same embedding as Table 1 which included contextual/semantic embeddings of either recurrent (LSTM) or transformer (sentence BERT) neural network architectures. For each embedding and each dataset, we then computed the Davies–Bouldin score (DBS), which is a ratio of within-cluster distance to between-cluster distance, where the two clusters were the cluster of relevant articles and cluster of irrelevant articles. Euclidean distance in tSNE-reduced 100-dimensional space was used. This DBS then became another input variable to explain the performance metrics. This DBS metric can be roughly interpreted as how distinct the overall text meaning of the relevant articles is from irrelevant ones. The smaller the number, the more distinct the relevant articles texts are from irrelevant ones.

The explanatory model took the form of an exponential function with three fitted (estimated) parameters:
(1)

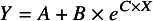



*X* and *Y*: the input and output variables respectively. In our case, *X* is one of the dataset characteristics (original full set size, relevant set size, relevance rate, DBS), while *Y* is one of the performance metrics (WSS @ 95%, MCC).

*A*: the horizontal asymptote or *y*-axis offset of the exponential. It sets the baseline of the exponential which roughly means performance for a set of articles with random inclusion (which theoretically should asymptote to zero but we allowed it to be estimated).

*B*: the starting point of the exponential and direction (whether it decays or grows). If it is nonzero, it also roughly means how much of the exponential component exists in explaining performance.

*C*: the rate of exponential decay (growth). It roughly means how quickly the performance degrades (rises) as dataset characteristic varies.

We considered other prediction forms, but they are expected to be underfitted (e.g., linear) or with too many degrees of freedom (e.g., splines) with generalizability being unlikely. An exponential function fulfilled our current needs for a compact, interpretable function that nonetheless has enough flexibility to demonstrate good fits. Model fitting and visualizations were done in Python 3.8.

## 3 Results

**Figure 1:**
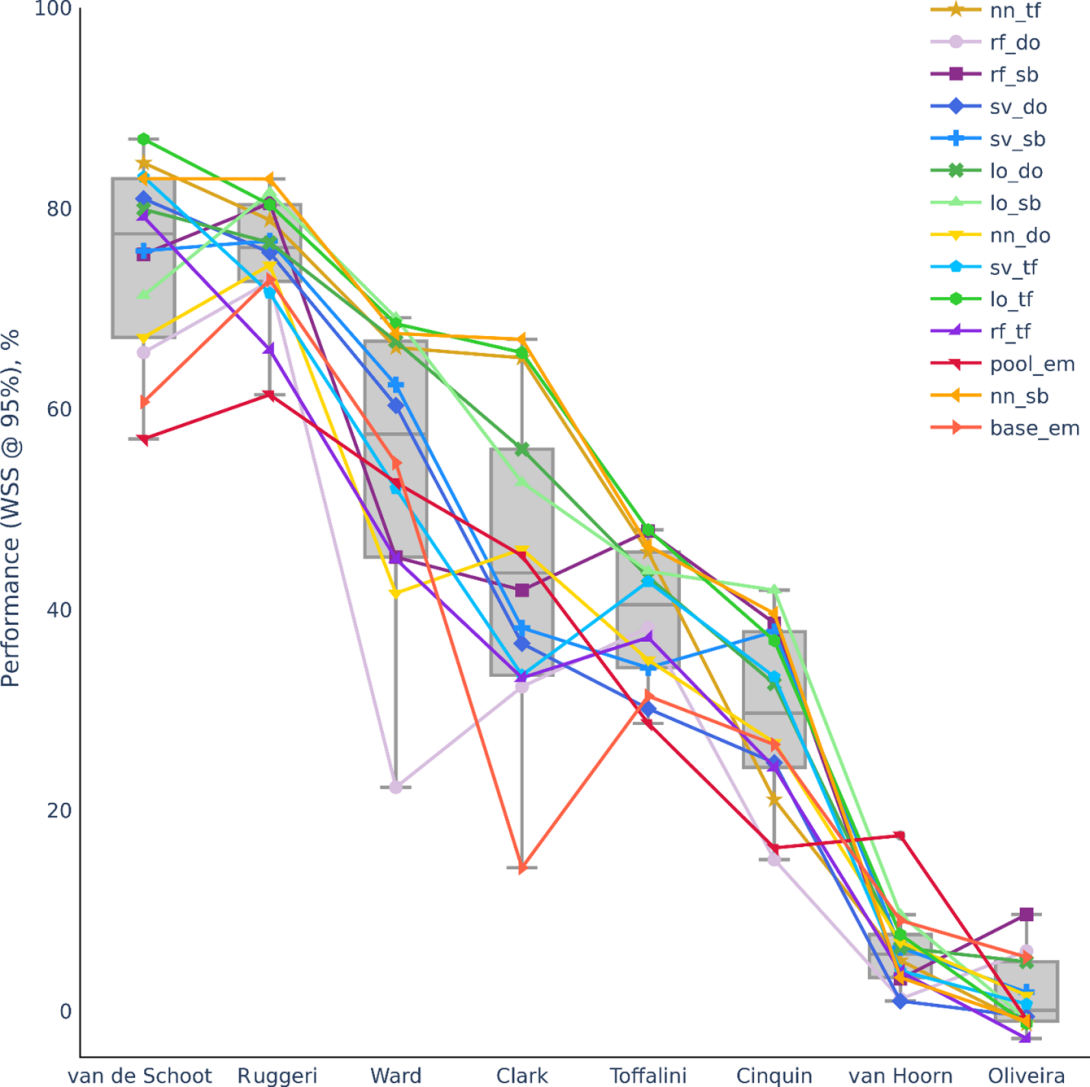
Performance metric (WSS @ 95%) for non-CNN models across all datasets. Refer to Section 2 and Supplementary Figure 1 for computation of metric. The higher the WSS @ 95%, the better ML screening is in terms of work saved. Datasets ordered based on median performance. For classifiers, rf = random forest, sv = support vector machine, lo = logistic regression, nn = 2-layer neural network, base = LSTM base, pool = LSTM with pooling. For feature extraction, do = doc2vec, sb = sentence BERT, em = embedding-LSTM, tf = TD-IDF.

Figure 1 plots the performance metric of WSS @ 95% for non-CNN family of models. Broadly speaking, the performance of ML screening varied greatly across datasets, ranging from 1.2–75.6% work saved with average of ~46%. We replicated the high 76.6% work saved for the benchmarking dataset.^32^ However, within each dataset, non-CNN models generally did not differ much in terms of performance, especially for Oliveira, van Hoorn, Toffalini, and Ruggeri datasets. Clark and Ward datasets were exceptions with large variations in performance across different non-CNN ML models. Non-CNN models are color-coded in Figure 1, and based on the colors, no single ML model seemed to be an outlier in having either the best or the worse performance across all datasets.

**Figure 2:**
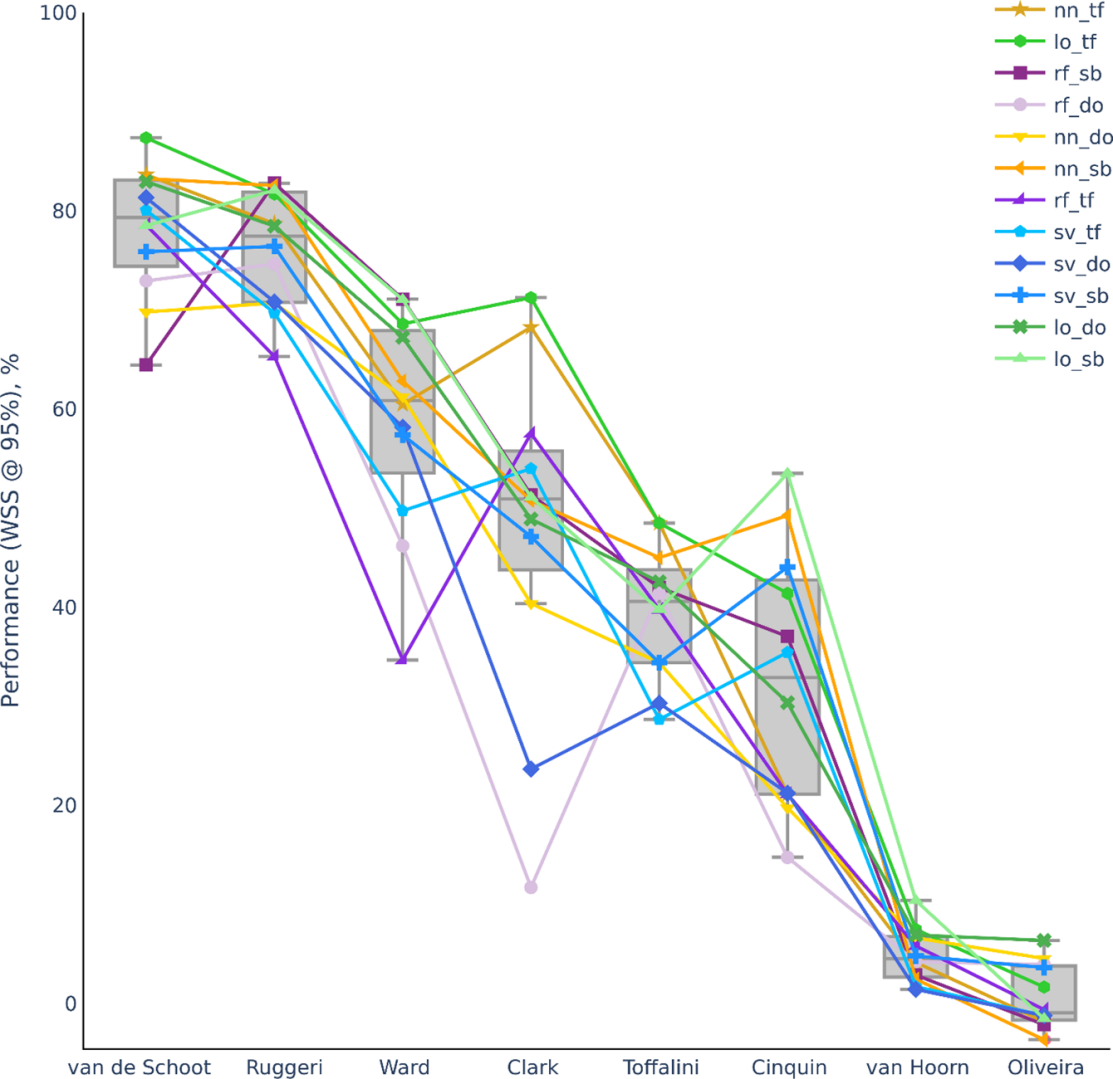
Performance metric (WSS @ 95%) for CNN models across all datasets. CNN models were switching models that started initially with manual feature extraction followed by shallow classifiers before switching to CNN models. Datasets ordered based on Figure 1 ordering. Refer to Section 2 for details. Same labels as Figure 1 for models preswitching.

Figure 2 plots the performance metric of WSS @ 95% for CNN models. The trends in Figure 2 were similar to non-CNN models in Figure 1. Correlation between dataset-specific average performance of non-CNN and CNN models was very high (*R* = 0.99). And CNN models work saved advantage over non-CNN models was marginal (+1.54% averaged across dataset). Different CNN models also did differ much in performance within a dataset except for Cinquin, Clark, and Ward.

**Figure 3:**
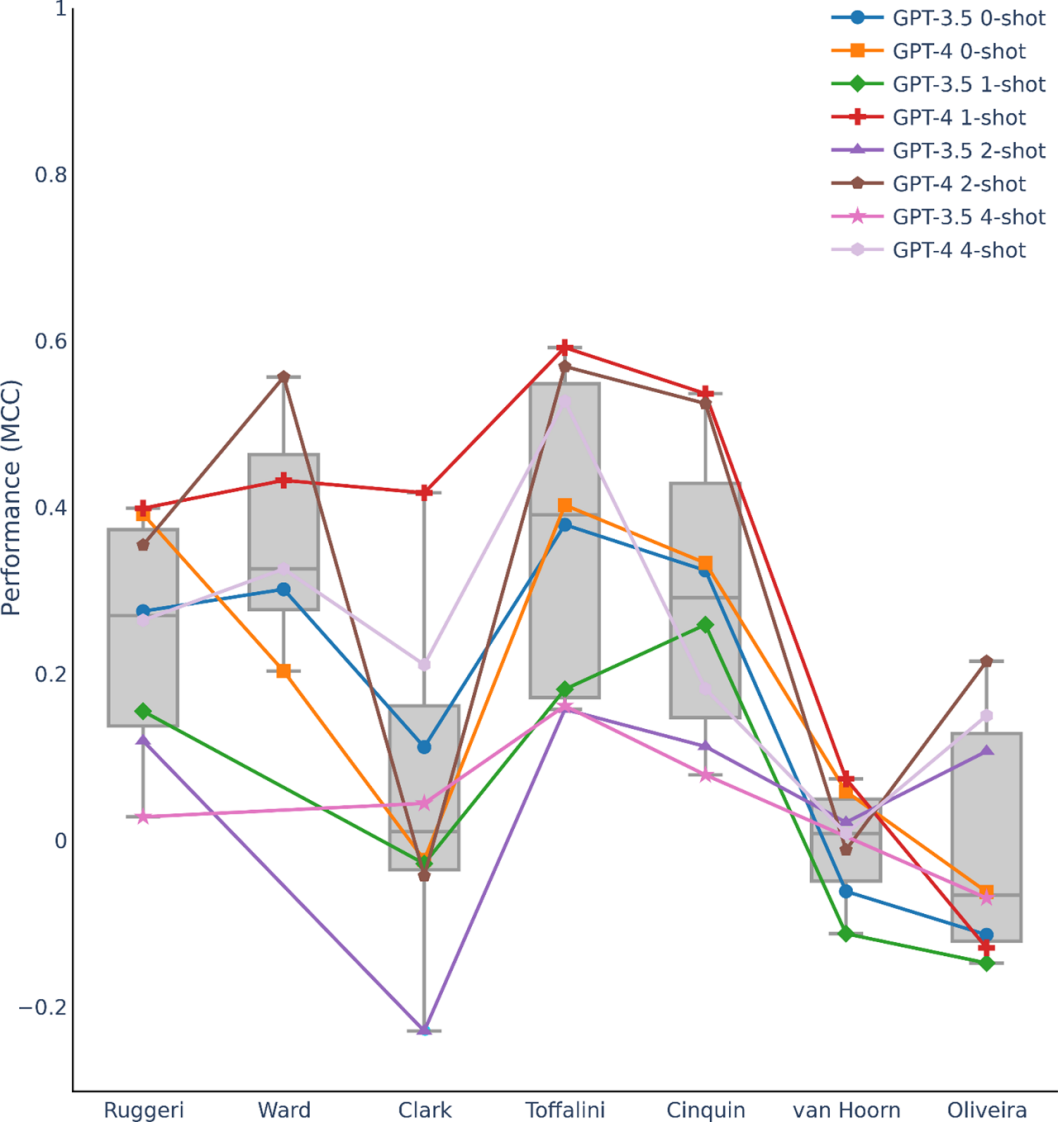
Performance metric (Matthew’s correlation coefficient [MCC]) for LLM prompt engineering. Datasets ordered based on Figure 1 ordering.

Figure 3 plots the performance metric of MCC for few-shot learning with GPT-3.5 and GPT-4 across the different datasets. Interestingly, the dataset-specific average performance for LLMs did not always match the classical and deep learning models in Figures 1 and 2. Classical and deep learning models performed well on the Clark and Ruggeri datasets but less so for LLMs relative to other datasets. Overall, LLM prompt engineering produced wide spread of performance ranging from MCC of ~0 (Clark and Oliveira datasets) to moderate performance of ~0.4 (Toffalini dataset).

**Figure 4:**
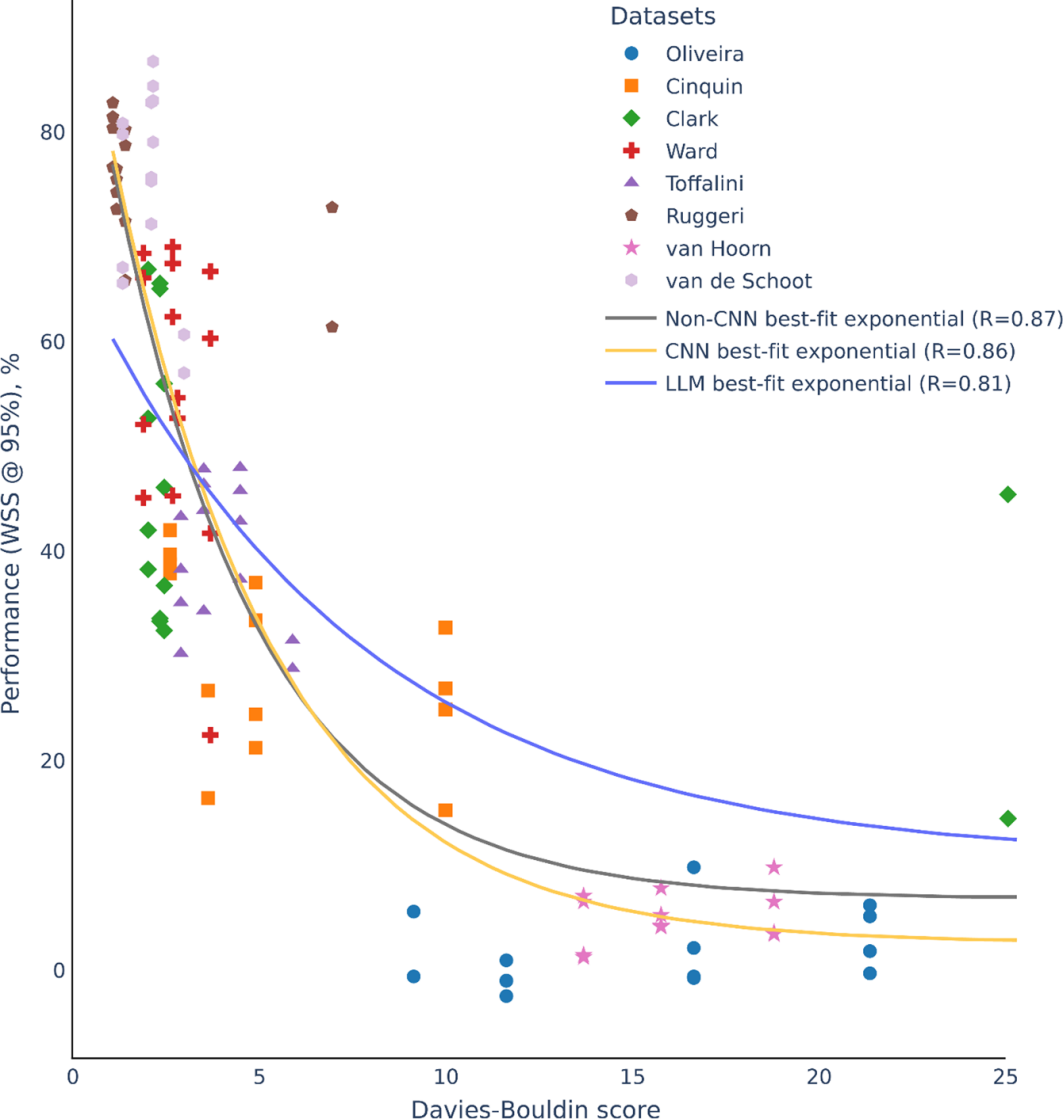
ML screening performance (WSS @ 95%) for non-CNN models as a function of separability of relevant and irrelevant article clusters as measured by DBS. Each dataset is made up of 14 datapoints due to the combination of embedding and classifier hyperparameters (see Table 2). Non-CNN best-fit exponential curve is the main curve fitted to the datapoints. The other curves are force-fitted to the datapoints using parameters from the other model families (CNN and LLM) to compare to the non-CNN best fit exponential curve. In general, parameters from the other model families can almost equally fit the datapoints, suggesting the universality of an exponential relationship explaining ML screening performance as a function of cluster separability regardless of model families.

**Figure 5:**
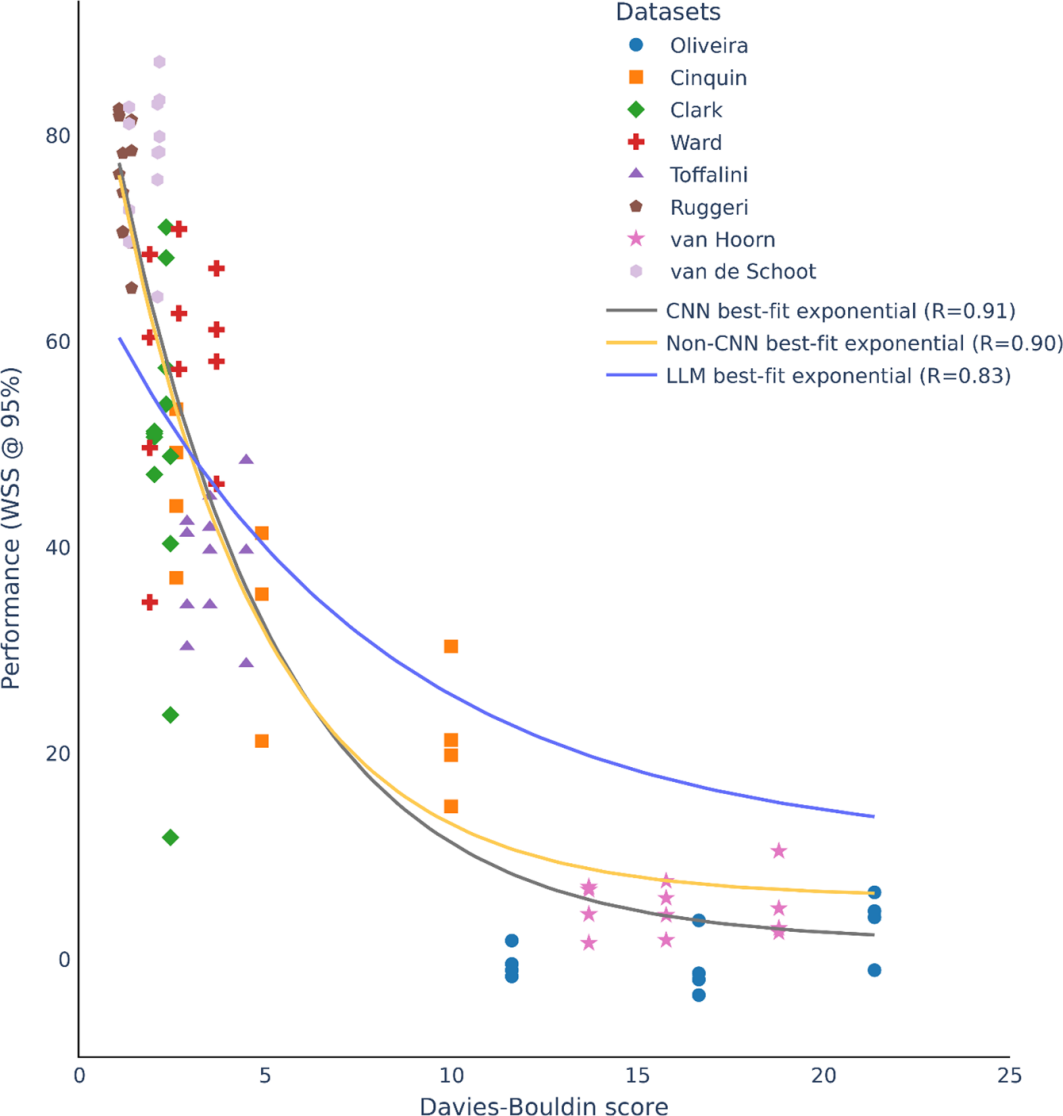
ML screening performance (WSS @ 95%) for CNN models as a function of separability of relevant and irrelevant article clusters as measured by DBS. Each dataset is made up of 12 datapoints due to the combination of starting models of embedding and classifier hyperparameters before switching to CNN (see Table 2). CNN best-fit exponential curve is the main curve fitted to the datapoints. The other curves are force-fitted to the datapoints using parameters from the other model families (non-CNN and LLM) to compare to the CNN best fit exponential curve. In general, parameters from the other model families can almost equally fit the datapoints, suggesting the universality of an exponential relationship explaining ML screening performance as a function of cluster separability regardless of model families.

**Figure 6:**
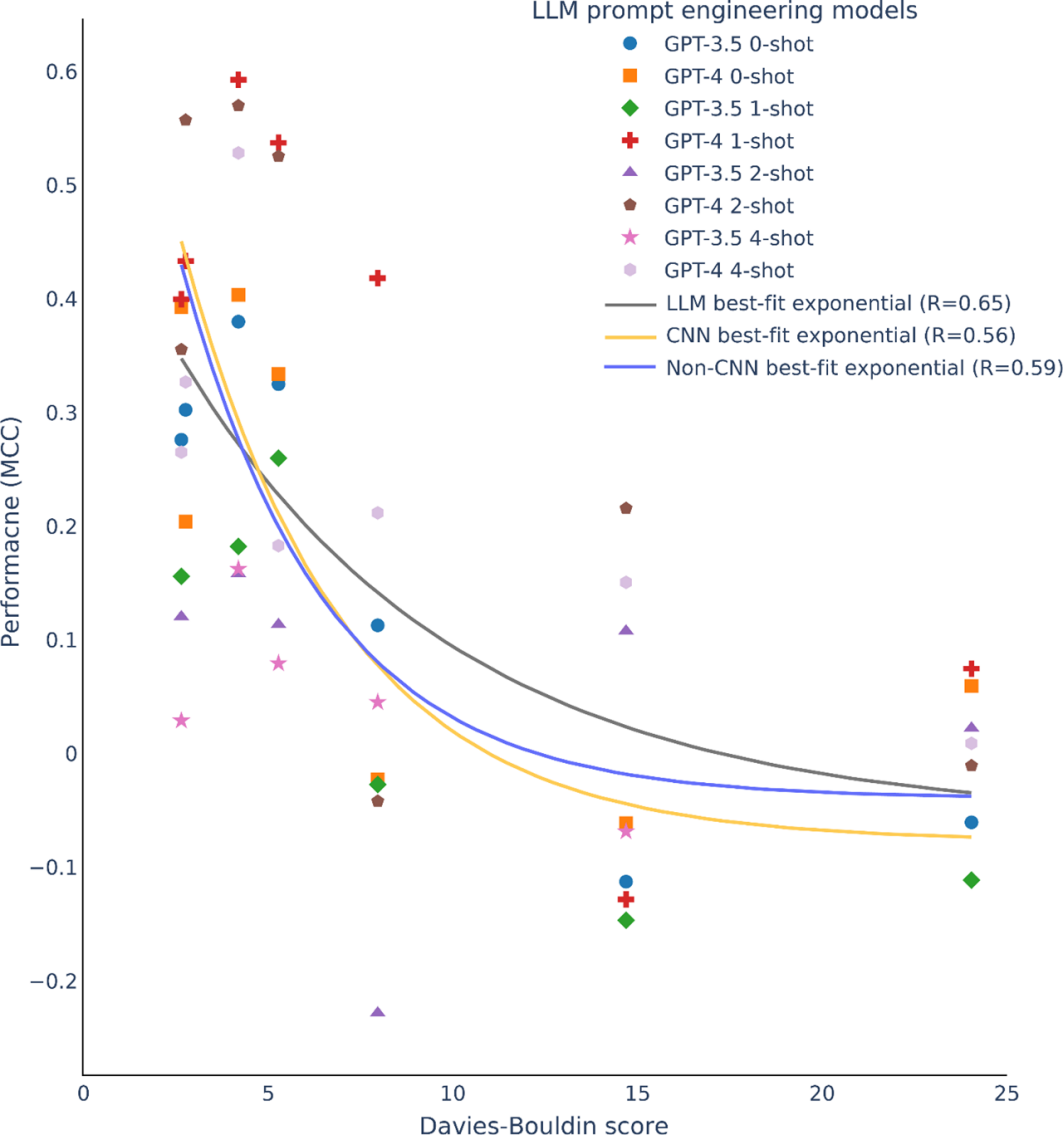
ML screening performance (MCC) for LLM prompt engineering models as a function of separability of relevant and irrelevant article clusters as measured by DBS. Because results varied across LLM models, we visually labeled the latter instead of datasets (like in Figures 4 and 5) to allow interpretation of LLM model trends. However, note that model fitting was done to datapoints from LLM models applied to all the datasets. LLM best-fit exponential curve is the main curve fitted to the datapoints. The other curves are force-fitted to the datapoints using parameters from the other model families (non-CNN and CNN) to compare to the LLM best fit exponential curve. In general, parameters from the other model families can almost equally fit the datapoints, suggesting the universality of an exponential relationship explaining ML screening performance as a function of cluster separability regardless of model families.

Given the extreme variation of ML screening performance, we searched for possible characteristics of the datasets that could explain the variation. Dataset characteristics such as full set size, relevant set size and relevance rate did not explain the performance variation well (*R* = 0.0–0.51; Supplementary Figures 2–4). Instead, we reasoned that the semantic representation of the articles might play an important role. When relevant articles are represented semantically as being very different from irrelevant articles, ML screening may have better performance. To pursue this idea, we examined the separability of clusters of relevant and irrelevant articles using various embedding. We discovered that cluster separability could indeed explain very well the variation in ML screening performance. Figures 4 and 5 show the WSS @ 95% performance metric as a function of cluster separability as measured by DBS. The lower the DBS, the lower the within-cluster distance relative to between-cluster distance, a measure of cluster separability. Datasets with very low DBS had very high ML model performance with an exponential function fitting the data very well for non-CNN (*R* = 0.87; Figure 4) and CNN models (*R* = 0.91; Figure 5). In fact, when we fitted the exponential function from non-CNN model datapoints to the CNN model datapoints and vice versa, the fits were still very high (CNN to non-CNN: 0.86; non-CNN to CNN: *R* = 0.90). For LLM prompt engineering, we observed a similar exponential trend though with reduced fit (Figure 6; *R* = 0.65). Nonetheless, the exponential function fitted from the non-CNN and CNN models to the LLM datapoints had similar fits (non-CNN to LLM: R = 0.59; CNN to LLM: 0.56). This cross-fitting across diverse model families suggests the universality of an exponential relationship explaining ML screening performance as a function of cluster separability. Based on the general equation form in ( 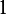 ), we averaged across CNN and non-CNN model families to obtain a more robust estimate to apply to DBS (input variable) and performance metric of WSS @ 95% (output variable):
(2)

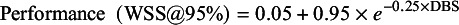

 where DBS is the cluster separability measured by Davies-Bouldin score. Based on this fitted formula, a useful heuristic emerges. For example, plugging in a DBS of 0.94 produces 80% WSS @ 95%, suggesting savings of 80% work at 95% recall. A DBS of 1 represents identical within-cluster distance to between-cluster distance; 0.94 suggests a slightly smaller within-cluster distance to between-cluster distance in order to save a lot of work. The fitted formula in ( 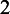 ) is general enough to estimate WSS for other DBS values (or vice versa).

## 4 Discussion

We evaluated a range of ML models from different families for ML screening of articles in actual systematic reviews in education. We found that if non-CNN models performed poorly on a specific dataset, CNN models were similarly poorly performing, suggesting limited variation within dataset, across models. However, across datasets, there was a large performance variation: some datasets were just “easier to automate” compared to others. LLM prompt engineering produced similarly wide performance variation across datasets. We found that this performance variation across datasets can largely be explained by separability of clusters of relevant versus irrelevant articles in high-dimensional embedding space.

Our results can be compared to two recent automated screening investigation related to education. In Chernikova, Stadler, Melev, and Fischer,^23^ ML models saved 70% of work while in Campos et al.,^22^ it was 58%. Our results were, on average, slightly lower at 46%. If we take a simple average of these results, it suggests that ML can help save about 58% of screening work in systematic reviews in education. Overall, this proportion of work saved is lower than the 91–64% work saved in six datasets across medicine, healthcare, and computer science.^24^ A recent preprint of a large-scale simulation study by Teijema, de Bruin, Bagheri, and van de Schoot^13^ found 62–71% of work saved for datasets from a large variety of fields. Campos et al.^22^ suggested that education may have characteristics that make it more challenging to automate screening. Educational research may not have standardized constructs like in other fields (e.g., derived from medical classification systems), and there is lack of conceptual clarity for many constructs, potentially making it more challenging for ML models to learn to clearly distinguish articles. Our results along with others in education may support this idea. However, we, and others, have found substantial variation in ML performance that tempers overgeneralizations of a single field and results may be more appropriately explained by dataset-specific limitations as discussed below.

Our study further extended previous studies including in education in two main ways. First, we tested a much larger number of ML models including deep learning-based CNN models which have not been evaluated in ML screening in educational research. In implementations on our datasets, we did not find big performance improvements by switching to CNN-based models (+1.54% performance improvement on average). Our results suggest that deep CNN layers are not always substantially better at extracting and learning complex, hierarchical text features used in systematic reviews. These results broadly agree with the findings of Teijema et al.^14^: when compared to suboptimal models, switching to CNNs can help but not when compared to already good performing models before switching. Furthermore, deep neural networks like CNNs have more complex architectures involving more decisions about hyperparameters. We used automated hyperparameter tuning just for number of hidden layers. Other hyperparameters could have been tuned (e.g., optimizer, activation function, etc.), but how much tuning can be done with highly imbalanced data is debatable. Overall, we propose that in cases where the best ML model for the dataset is not clear, switching to deep neural networks can help, especially when there is a need to identify any and all remaining most difficult last records.^14^ However, if there is a likely optimal model based on prior knowledge of the domain or nature of dataset, CNNs may not be worth the complexity, time, and resource use.

Second, our study systematically examined the potential for LLM prompt engineering in automating the review process. Based on our crafted prompts and shots, we obtained overall performance (computed via MCC) of ~0.4. This result suggests the potential for LLMs to sensitively and precisely identify articles for systematic reviews. However, the performance was still not close to being high enough for researchers to be confident of its sole use. Moreover, LLMs may not be able to clearly explain and justify why certain articles were selected or not which may not satisfy transparency requirements of systematic reviews. However, LLMs can still play a lower-stakes role such as a second reviewer to pair up with a human reviewer. Alternatively, when prompted to be more liberal in detection, they may uncover additional articles for humans to further screen for final decision, that is, enhance sensitivity at the expense of precision. In other words, while LLMs may not be able to fully replace human reviewers in the screening process, they may still play a role in the screening process.

In their large-scale study of ML screening models, Teijema et al.^13^ argued for research to better predict model performance as “currently, we don’t fully understand which dataset characteristics influence the performance of a model” (p. 24). Based on our results, we propose that semantic distinctiveness of clusters of articles can explain ML performance. We found that embedding article titles and abstracts into high dimensional space can reveal clusters whose separability can strongly predict automated screening performance across diverse families of models. When a dataset has a cluster of relevant articles that is clearly distinct from the cluster of irrelevant articles (as measured by intra- to inter-cluster distance), ML performance tends to be very high, leading to high work savings. On the one hand, this result may seem obvious since embedding is indeed the first step for providing features that downstream classifiers then work on. Embedding that provides high separability can then lead to better classification. On the other hand, it is still unexpected that the exponential relationship relating performance to embedded cluster separability is a strong one that applies to diverse model families including to LLM prompt engineering (where the transformer-based embedding is quite different from the ones being employed here for the non-CNN and CNN models here). Our exponential fits provide both an understanding of performance of ML screening as well as heuristic for practical use.

We foresee multiple roles of our heuristic in the screening pipeline. In one pipeline, cluster separability measures (e.g., DBS) can provide an early indication to the screeners as to whether applying ML models can help speed up the rest of the screening process. And if they can, what kind of models may be better at speeding up the screening, that is, inform model selection. In another pipeline, cluster separability measures may be helpful in iterating the search and screening criteria. When the initial set of articles being screened produce poor separation in clusters of relevant and irrelevant articles, the researchers may consider refining the inclusion and exclusion criteria and/or potentially even modify the search terms. For example, when cluster separability is low in an initial search with a subset of labelled articles, a stricter set of screening criteria may be set to further distinguish the relevant from the irrelevant articles. Alternatively, the subsequent search terms may need to be refined to produce a narrower set of returns, making the review much more focused. In other words, the cluster separability measure can inform management of different priorities (effort versus scope).

In another pipeline, cluster separability can play a role in the termination of screening process, oftentimes called the stopping decision. In ML screening via active learning, a decision has to be made as to when to continue screening the remaining articles. Different criteria balancing tradeoffs of recall with additional effort have been proposed as reviewed in the study by Callaghan and Müller-Hansen.^37^ Our cluster separability heuristic provides yet another tool to aid in the stopping decision: datasets that have high cluster separability of the already screened articles can be confidently terminated without sacrificing much recall, if at all. The cluster separability heuristic can also adaptively change the stopping criteria. Low cluster separability may require more conservative stopping criteria to ensure high recall rates at the expense of more effort but high cluster separability can likely tolerate liberal stopping criteria. In summary, our work suggests different ways the cluster separability heuristic can be incorporated into various ML screening pipelines. Equation ( 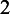 ) with the robust, cross-model family estimates can be considered for future research and/or default software setting.

Our study has numerous limitations. First, it is somewhat limited in the number of datasets although they do provide large variations that are useful for modeling. Future research will have to test whether our results, especially exponential function of ML performance against cluster separability, would apply more generally. Second, our LLM prompt engineering is still exploratory. Although GPT-3.5 and GPT-4 are currently quite powerful, we did not test an extensive set of LLMs including those that are fine-tuned for specific fields^38^ (however, to our knowledge, there is none so far for educational research). Third, our datapoints were obtained from multiple runs that averaged across the noise withing a dataset; thus, our estimates of fits may be overly optimistic when applied to actual screening which typically does not involve averaging from multiple model runs. Finally, the article screening stage is but one early part of the systematic review process. There are other time-consuming stages such as full-text reading, data extraction, and visualization, all of which can potentially be automated by ML^39^ but that we did not address in our study.

## Supporting information

Ali et al. supplementary material Ali et al. supplementary material

## Data Availability

Code and datasets are available at https://doi.org/10.25340/R4/VB85L1.

## Supplementary material

To view supplementary material for this article, please visit http://doi.org/10.1017/rsm.2024.16.
