# Supplementary material for: Can machine learning help accelerate article screening for systematic reviews? Yes, when article separability in embedding space is high
Source: Res Synth Methods. 2025 Mar 10;16(1):194–210. doi: 10.1017/rsm.2024.16 (PMC12621506; doi:10.1017/rsm.2024.16)
Supplement: Ali et al. supplementary material [file S1759287924000164sup001.docx]

**Supplementary Information**

**Can machine learning help accelerate article screening for systematic reviews?**

**Yes, when article separability in embedding space is high**

Farhan Ali, Amanda Swee-Ching Tan, Serena Jun-Wei Wang


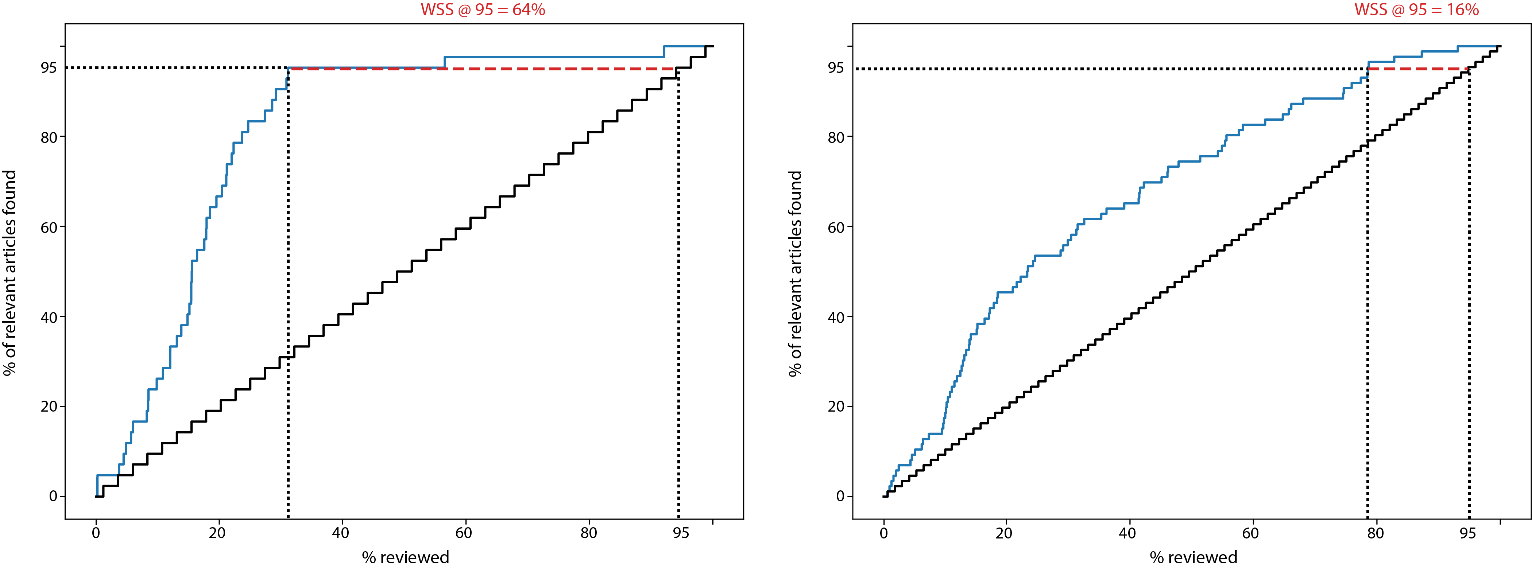


Supplementary Figure 1 Graphical illustration of metric of WSS @ 95%. Left, an example of high WSS @ 95% value of 64%, indicating that 64% of work was saved compared to random sampling. Right, example of low WSS @ 95% value of 16%, indicating only 16% of work was saved compared to random sampling.


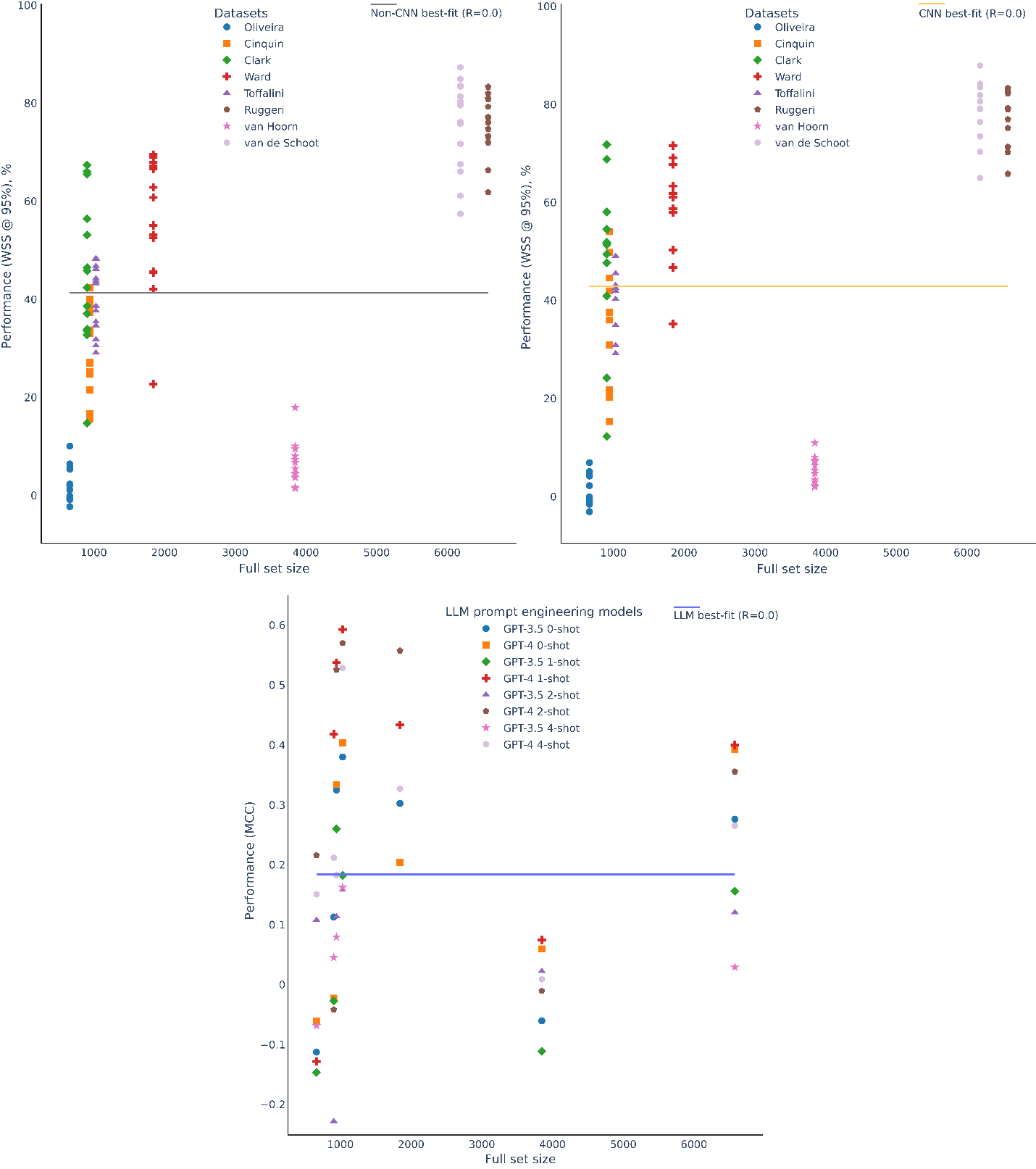


Supplementary Figure 2 ML screening performance for non-CNN (top left), CNN (top right), and LLM prompt engineering (bottom) models as a function of full set size. This supplementary figure can be compared to the main Figures 4, 5, and 6.


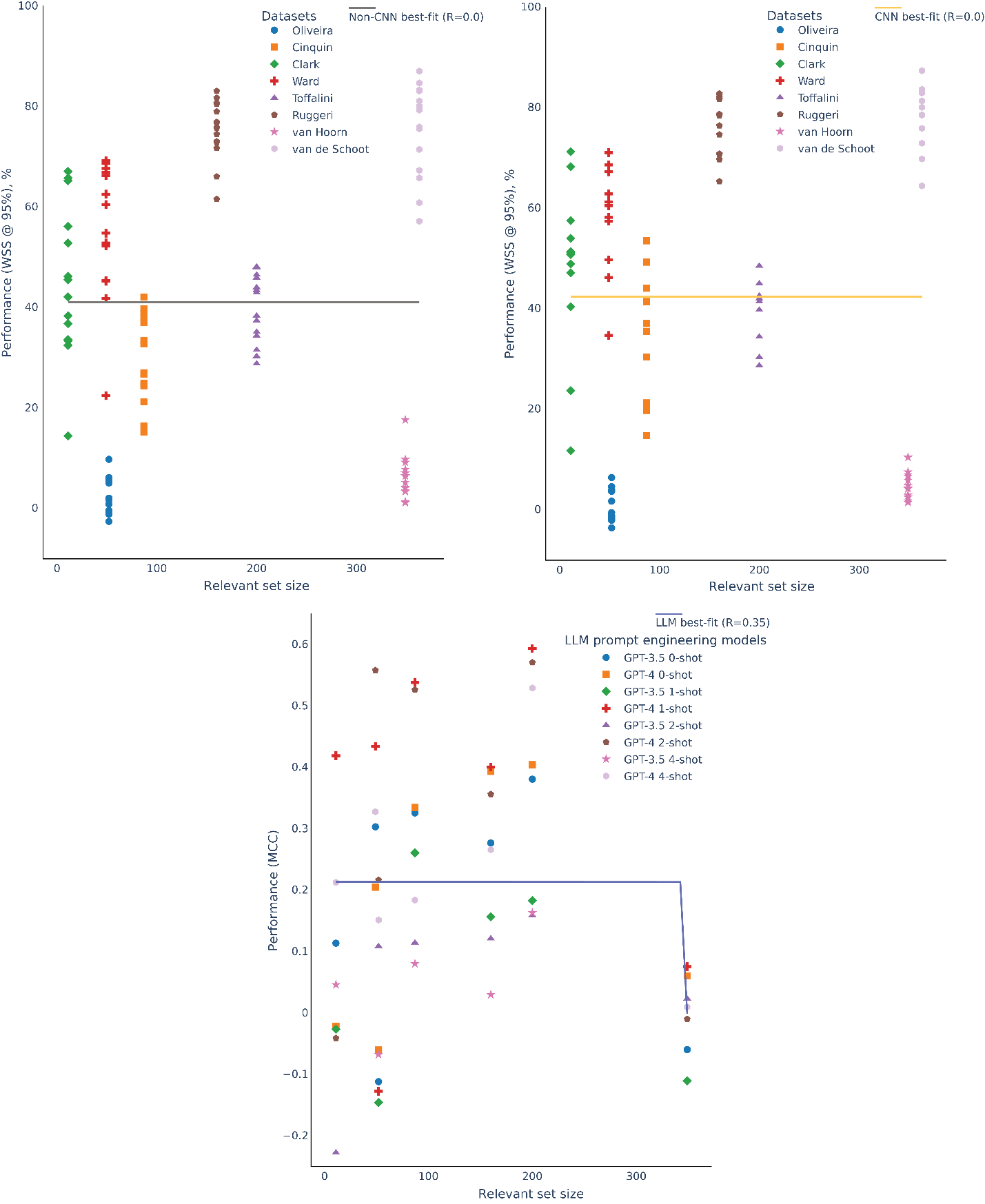


Supplementary Figure 3 ML screening performance for non-CNN (top left), CNN (top right), and LLM prompt engineering (bottom) models as a function of relevant set size. This supplementary figure can be compared to the main Figures 4, 5, and 6.


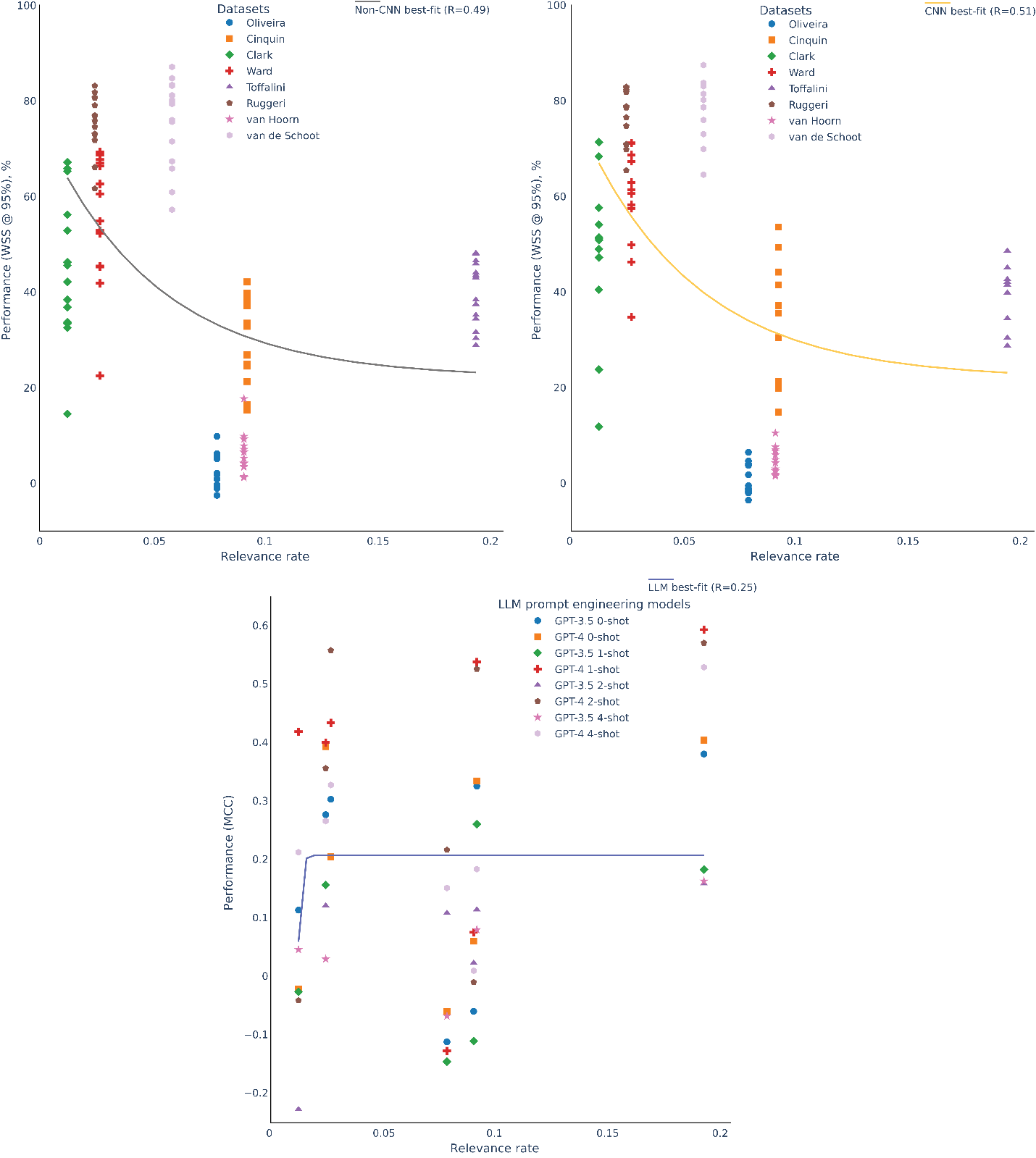


Supplementary Figure 4 ML screening performance for non-CNN (top left), CNN (top right), and LLM prompt engineering (bottom) models as a function of relevance rate. This supplementary figure can be compared to the main Figures 4, 5, and 6.

Supplementary Table 1 LLM prompts for few-shot learning

| Cinquin |
| --- |
| You are a researcher. You are screening research articles for a systematic review on online e-learning and cognitive disabilities. Based on the title and abstract of each research article, decide on whether the article will be included in the systematic review based on the following inclusion criteria: 1. online e-learning accessibility for individuals with cognitive impairment. E-learning is defined as e-learning as instruction delivered via a digital device intended to support learning; 2. Studies that explicitly refer to client/server architecture such as the web; 3. Proposed guidelines or a framework, and those with a purpose of intervention instead of instruction if they explicitly expected a possible transfer for learning; 4. studies that explicitly refer to people with cognitive impairments exclusively (for instance, studies focusing on people with attention disorders) or people with cognitive impairments along with other impairments (studies not focusing on a specific disability but considering cognitive impairments). These are the exclusion criteria: 1. Not peer-reviewed scientific articles (book chapters, summaries of proceedings); 2. Non-English language articles. |
| Clark |
| You are a researcher. You are screening research articles for a systematic review on orthographic support for word learning in clinical populations. Based on the title and abstract of each research article, decide on whether the article will be included in the systematic review based on the following inclusion criteria: 1. group studies, randomized controlled trials, single  case experimental design studies, case series, and/or multiple case studies; 2. participants under age 18 years, (b) participants belonging to a clinical category (i.e., developmental language disorders (DLD), autism spectrum disorder (ASD), Down syndrome, dyslexia, intellectual disability, hearing impairment, or cerebral palsy); 3. outcomes that include word learning, (d) an experimental or quasi-experimental design; 4. publication in a peer-reviewed journal, and (f ) orthography as a manipulated condition. These are the exclusion criteria: 1. Outcome measures solely targeting reading or spelling ability; 2. Adult populations. |
| Oliveira |
| You are a researcher. You are screening research articles for a systematic review on social and emotional learning (SEL) interventions for teachers. Based on the title and abstract of each research article, decide on whether the article will be included in the systematic review based on the following inclusion criteria: 1. An empirical study with quasi-experimental or experimental designs on the efficacy of a SEL intervention for in-service preK-12 teachers in their personal and/or occupational outcomes; 2. Sufficient information reported to calculate the effect sizes of the interventions’ impacts; 3. Published in peer-reviewed journals. These are the exclusion criteria: 1. Papers targeting university and/or pre-service teachers; 2. Did not access impacts on teacher-level variables. |
| Ruggeri |
| You are a researcher. You are screening research articles for a systematic review on motor and physical activity intervention in children with autism spectrum disorder (ASD). Based on the title and abstract of each research article, decide on whether the article will be included in the systematic review based on the following inclusion criteria: 1. Group study designs included cohort studies and clinical trials; 2. Participants included were children with ASD from birth to 21 years, and if other diagnoses were included, the results of participants with ASD were statistically analyzed separately; 3. All types of motor and physical activity interventions. (4) Motor outcome of body structure and function, activity, or societal participation was measured using an objective outcome measure and statistically analyzed; (5) Published in English; and (6) The study investigated either (a) the effects of a motor intervention on a motor outcome or (b) the effects of a motor learning variable on motor skill acquisition, transfer, and/or retention. These are the exclusion criteria: 1. Unpublished papers such as abstracts, conference proceedings, and dissertations. 2. Studies published before 2000. |
| Toffalini |
| You are a researcher. You are screening research articles for a systematic review on treatments for dyslexia. Based on the title and abstract of each research article, decide on whether the article will be included in the systematic review based on the following inclusion criteria: 1. Reporting quantitative data concerning treatment efficacy on individuals with dyslexia/reading disorder 2. Treatment approach can be of any type, but it must aim to improve reading performance as its ultimate goal; 3. Written in English or Spanish, French, Italian, Portuguese, and Hungarian; 4. Participants must either be clinically diagnosed with developmental dyslexia (or reading disability or reading disorder) or having a profile compatible with a reading disorder as reported by the authors; in the latter case, participants must have reading performance either below the 25th percentile or one standard deviation below the population mean as assessed using standardized tests in their mother tongue; 5. Participants must be described as having normal intelligence or an IQ not below 70 (if reported); 6. Any co-morbidity or co-occurring condition is acceptable, but they must be compatible with dyslexia status; 7. Include at least one control group comprising individuals with dyslexia, who must either be untreated, waiting list, or active control (e.g., a placebo condition; no comparisons between alternative/competing treatments were considered); 8. Group allocation must be randomized; however, studies which did not explicitly mention whether the allocation was randomized were still included. 9. Participants’ reading ability must be assessed at least twice, including before (pre-test score) and after treatment (post-test score). These are the exclusion criteria: 1. Deafness, neurological conditions, intellectual disability in one or more participants, or low socioeconomic status as a predominant condition of the entire sample. |
| van Hoorn |
| You are a researcher. You are screening research articles for a systematic review on risk factors in early life for developmental coordination disorder. Based on the title and abstract of each research article, decide on whether the article will be included in the systematic review based on the following inclusion criteria: 1. Address children with developmental coordination disorder (DCD): children who met the diagnostic criteria for DCD according to the Diagnostic and Statistical Manual of Mental Disorders (DSM), Fourth or Fifth Editions, or children with motor impairment as assessed with a standardized motor test (e.g. Movement Assessment Battery for Children [MABC] score below the 16th centile as a cut-off) or another appropriate, valid, reliable, and standardized motor test (appropriately norm-referenced). Also, articles in which children with probable DCD were identified by means of questionnaires like the DCD Questionnaire were included; 2. Participants’ mean age had to be between 5 and 13 years; 3. Studies that addressed the follow-up of specific groups of infants at increased risk of neurodevelopmental disorders, e.g. infants with neonatal hypoxic ischaemic encephalopathy or infants born preterm with a lesion of the brain but no diagnosis of cerebral palsy are eligible; 4. Studies addressed associations between DCD (or motor impairment) and early life factors (i.e. pregnancy-related factors, birth factors, child factors, or sociodemographic determinants). Early life factors were defined as factors occurring in the period ranging from pregnancy until 3 months post-term, since the latter age is characterized by a major neurodevelopmental transition. These are the exclusion criteria: 1. Included fewer than 10 participants; 2. Reviews or case-reports; 3. Studies specifically addressing the effect of drugs (e.g. caffeine) or nutritional supplements (e.g. vitamin D and vitamin A). 4. Children with a neurological disorder such as CP. |
| Ward |
| You are a researcher. You are screening research articles for a systematic review on ADHD teacher training programs. Based on the title and abstract of each research article, decide on whether the article will be included in the systematic review based on the following inclusion criteria: 1. Populations are either teachers or children. For teachers, primary or secondary school teachers. For children, a diagnosis of attention-deficit/hyperactivity disorder (ADHD) or identified as displaying ADHD-type behaviors (i.e., hyperactivity, impulsivity, inattention/ off-task behavior) or children in primary or secondary mainstream education (aged 4–16 years); 2. For intervention type, ADHD teacher training interventions for in-service teachers of any type, delivery mode, duration or intensity or ADHD teacher training interventions which have one condition as teacher training only; 3. For comparison, either no comparison group or waitlist control, alternative treatment, control group; 4. For outcomes of teachers in mainstream primary and secondary classrooms: measures of teachers’ ADHD knowledge or measures of teachers’ behavior management strategies toward children with ADHD and ADHD-type behaviors. For outcomes of children with a diagnosis of ADHD or identified as displaying ADHD-type behaviors in primary or secondary education: measures of child ADHD symptoms (e.g., inattention including off-task behaviors, impulsivity, hyperactivity) and related impairments, including problem behaviors and social functioning; 5. Study must be controlled trials (randomized and non-randomized) or intervention studies; 6. Peer-reviewed journal articles and gray literature (dissertation, theses, reports, articles in press). These are the exclusion criteria: 1. For teacher population, pre-school teachers, post-compulsory, education teachers, teaching assistants, other educational professionals, teachers in special schools. For student population, children in special schools, children in preschool or post-16 education; 2. For intervention type, the following are excluded: teacher training interventions delivered prior to teacher qualification for example, in teacher training colleges; or training interventions where the teacher component is combined with other groups for example, parents, child; or training interventions where ADHD is a minor component of the training, for example, induction training, or one part of a larger training programme; 3. For outcomes, any measures for special education teachers children in special schools, pre-school or post-16 education. 4. Qualitative studies; 5. Conference papers. |
